# Duration of immunity against heterologous porcine parvovirus 1 challenge in gilts immunized with a novel subunit vaccine based on the viral protein 2

**DOI:** 10.1186/s12917-020-02394-4

**Published:** 2020-06-09

**Authors:** Beatriz Garcia-Morante, Marta Noguera, Sonja Klocke, Kathrin Sommer, Philip Bridger

**Affiliations:** 1 Centcinc, C/Montserrat de Casanovas 105, 08032 Barcelona, Spain; 2 Boehringer Ingelheim Veterinary Research Center GmbH & Co. KG, Bemeroder Straβe 31, 30559 Hannover, Germany; 3 Boehringer Ingelheim Vetmedica GmbH, Binger Straβe 173, 55216 Ingelheim, Germany

**Keywords:** Porcine parvovirus, Subunit vaccine, Vaccine efficacy, Duration of immunity, Mass vaccination, Pigs

## Abstract

**Background:**

Porcine parvovirus 1 (PPV1) is widespread in commercial pig farms worldwide and has a significant impact to the swine industry. Long-lasting immunity achieved by means of vaccination is the main tool to prevent PPV1 infection and its associated clinical signs. Here we evaluated the duration of immunity (DOI) conferred by a novel subunit vaccine based on the viral protein (VP) 2 of PPV1, named ReproCyc® ParvoFLEX. The DOI was assessed at 6 months post-vaccination following the standard vaccination scheme (phase I) or after re-vaccination (phase II) with a single injection administered 24 weeks after the basic vaccination scheme. A total of 46, five to six-month-old gilts, free of PPV1 and porcine reproductive and respiratory syndrome virus (PRRSV), were randomly assigned to 6 groups (three in each phase): the negative control groups were inoculated with sodium chloride (NaCl), the vaccinated groups were immunized with the PPV1 subunit vaccine and the strict controls were neither treated nor challenged. Subsequently, the negative control and vaccinated groups from each phase were challenged with a heterologous PPV1 strain. Infection of fetuses was the primary outcome parameter for efficacy, though other supportive parameters were PPV1 viremia and serological status of the gilts and the condition of their fetuses (i.e. normal, autolytic, or mummified).

**Results:**

All gilts vaccinated against PPV1 tested seropositive at challenge and viremia after challenge was detectable only in the non-vaccinated animals. In this regard, fetuses positive to PPV1 by PCR were only found in litters from non-vaccinated sows.

**Conclusions:**

These results point out that the immunity developed by the PPV1 subunit vaccine is effective in terms of preventing viremia, transplacental infection of fetuses and fetal death caused by PPV1 infection. ReproCyc® ParvoFLEX was demonstrated to protect fetuses against heterologous PPV1 challenge with a DOI of 6 months after vaccination.

## Background

Porcine parvovirus genotype 1 (PPV1) is recognized as a ubiquitous infectious cause of reproductive failure in swine worldwide [1]. The stage of gestation at which infection occurs is the determinant for clinical disease manifestations, which are mainly described by the acronym of SMEDI (stillbirth, mummification, embryonic death, and infertility). In general, the infected gilts or sows themselves do not show clinical signs, and virus transmission to the fetuses is prompt to occur if the dam is not immunized [1]. Therefore, to maintain herd immunity against PPV1 is an imperative goal to prevent reproductive failure associated to the virus and the reason why vaccines are routinely used in breeding herds [2]. Long-term vaccination programs are regarded as cost effective methods for controlling PPV1-induced reproductive failure in pig herds suffering endemic and epidemic PPV1 infection [3].

Porcine parvovirus 1 is a small, single-stranded DNA virus with a non-enveloped capsid that belongs to the species *Ungulate protoparvovirus* 1 in the genus *Protoparvovirus* [2, 4]. The capsid of PPV1 is a spherical shell consisting of 60 copies of a mixture of structural viral proteins (VPs) 1 and 2 arranged in icosahedral symmetry [5]. These VPs differ only in their amino-terminal initiation or post-translational modification; PPV1 has 729 amino acid residues in VP1, of which 150 form the amino-terminal unique portion which is absent in VP2 [6]. Notably, VP2 is the major capsid protein of PPV1 and is the main target of neutralizing antibodies against PPV1, which are a decisive factor in the outcome of PPV1 infection [6–9]. Therefore, VP2-based vaccines continue to be the focus of PPV1 vaccine research [10, 11].

New PPV1 capsid profiles with different amino acid patterns and distinct antigenic properties have been described, including differences in cross-neutralization of the sera raised against recent field isolates from Germany and viruses used in commercial vaccines [7, 9]. These findings have led to the hypothesis that the emergence of new capsid profiles could be due to viral adaptation to the broadly used vaccines and therefore “old” PPV1 vaccine strains may not effectively neutralize these “new” viruses. In consequence, a need for updated PPV1 vaccines conferring rapid, long-lasting as well as effective immunity against a broad range of heterologous viral strains has been suggested [4]. ReproCyc® ParvoFLEX (Boehringer Ingelheim Vetmedica GmbH, Ingelheim am Rhein, Germany) is a recently licensed subunit vaccine for pigs based on a recombinant baculoviral expression system producing the protective antigen VP2 of PPV1.

In order to determine whether the aforesaid novel PPV1 subunit vaccine provides long-term immunity to PPV1 challenge in target animals, a randomized, blinded, negative controlled vaccination challenge study conducted according to the requirements described in *European Pharmacopoeia* (Ph. Eur.; monograph 01/2017:0965) and the principles of Good Clinical Practice (GCP), was carried out. The study sought to evaluate the duration of immunity (DOI) of: (i) the basic PPV1 vaccination scheme and (ii) the re-vaccination when a single shot is administered 6 months after the basic vaccination scheme.

## Results

### Animals excluded from the study

The present study was organized in two phases (Table 1). Phase I evaluated the DOI of the basic PPV1 vaccination scheme (i.e. 6 months after a two-dose regimen) and phase II assessed the DOI of the re-vaccination (i.e. 6 months after a single boost immunization). Once accomplished the vaccination scheme in each phase (i.e. study day [SD] 21 in phase I and SD 202 in phase II), all female pigs were estrus synchronized and artificially inseminated. Apart from the strict controls (groups 3-SC and 6-SC), animals from the negative control (1-NC and 4-NC) and vaccinated (2-Vac and 5-Vac) groups were challenged at day 40 (± 1) of gestation (SD 202 in phase I and SD 385 in phase II). Necropsy was performed at day 90 (± 1) of gestation, when vaccine efficacy was evaluated. Overall, 6 challenged gilts had to be excluded before the study was completed; they were not assessed for efficacy because they were found to be not pregnant at necropsy. Pregnancy checks were performed from day 30 to 35 of gestation, and these gilts were marked as not diagnosable (i.e. pregnancy diagnosis could not be clearly stablished). Since PPV1 challenge was scheduled at day 40 (± 1) of gestation, these gilts were challenged anyway. They were used to assess serology and viremia, though no fetal evaluation could be performed afterwards. The distribution of the excluded animals through the groups is shown in Table 2.

**Table 1:** Study design. A total of 46 gilts were randomized into 6 groups and enrolled into the study performed in two phases. Animals from phase I (basic vaccination scheme) were challenged at SD 202 whereas animals from phase II (re-vaccination scheme) were challenged at SD 385. The study ended at 90 days of gestation of the gilts, when their fetuses were collected for evaluation

	Group	No. of animals		1st treatment	2nd treatment	3rd treatment	Challenge	Evaluation of fetuses
			Study day	0	21	–	202	≈252
			Gestation day	–	–	–	≈40	≈90
Phase I	1-NC	12		NaCl	NaCl	–	Yes	Yes
	2-Vac	11		Vaccine	Vaccine	–	Yes	Yes
	3-SC	4		–	–	–	No	No
				**1st treatment**	**2nd treatment**	**3rd treatment**	**Challenge**	**Evaluation of fetuses**
	**Group**	**No. of animals**	**Study day**	0	21	202	385	≈435
			**Gestation day**	–	–	–	≈40	≈90
Phase II	4-NC	8		NaCl	NaCl	NaCl	Yes	Yes
	5-Vac	7		Vaccine	Vaccine	Vaccine	Yes	Yes
	6-SC	4		–	–	–	No	No

*NC* negative control; *Vac* vaccinated animals with the PPV1 subunit vaccine; *SC* strict control

**Table 2:** Number (No.) of animals at the distinct phases of the study. From the initial number of animals included (*n* = 46), 32 animals were evaluated for efficacy

	Group	No. of animals included	No. of animals evaluated for efficacy
Phase I	1-NC	12	8
	2-Vac	11	10
	3-SC^a^	4	–
Phase II	4-NC	8	8
	5-Vac	7	6
	6-SC	4	–

*NC* negative control; *Vac* vaccinated animals with the PPV1 subunit vaccine; *SC* strict control

^a^Two animals died before phase I challenge of their counterparts from groups 1-NC and 2-Vac

### Gilts serology and viremia

**Fig. 1:**
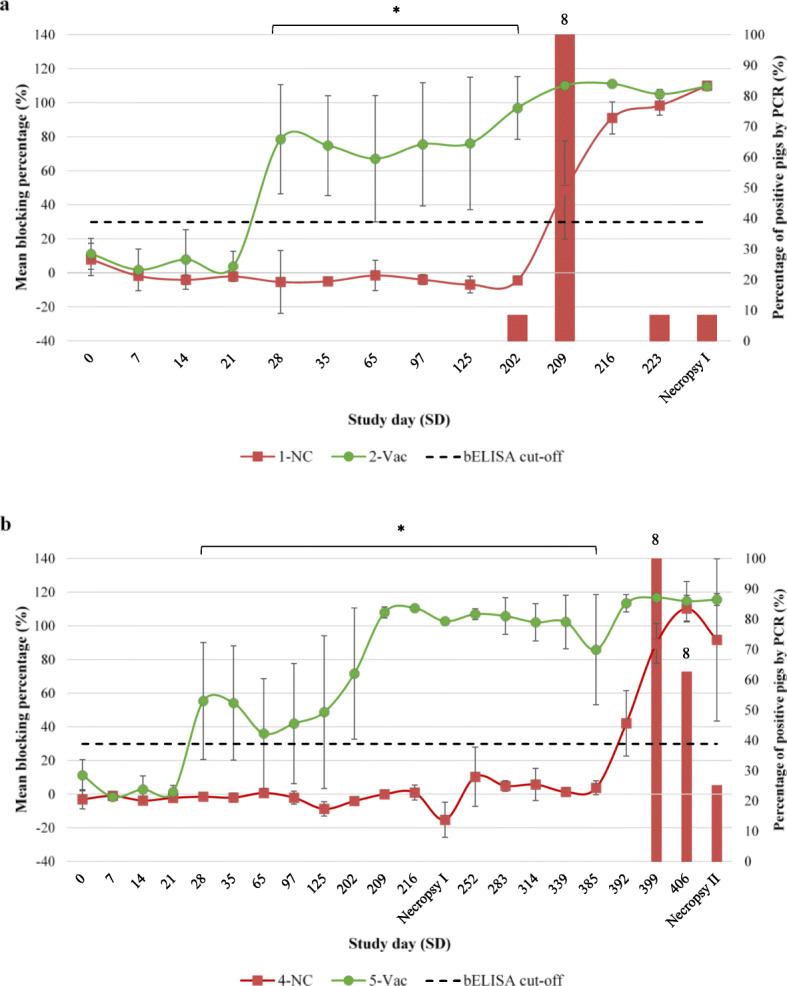
Serology and viremia results in female pigs. Mean (± standard deviation) blocking percentage (bELISA; lines) and percentage of viremic animals (PCR; columns) to PPV1 in groups 1-NC and 4-NC (red) and groups 2-Vac and 5-Vac (green) in phase I (**a**) and phase II (**b**). Challenge was performed on SD 202 on phase I and on SD 385 in phase II. Discontinuous lines represent the seropositivity threshold. No data of PPV1 detection by PCR in vaccinated groups is shown, as no viremia was detected throughout the study. Nevertheless, statistical differences are represented. *Statistical differences (*p* < 0.05) in mean blocking percentages between groups. ☐Statistical differences (*p* < 0.05) in percentages of viremic gilts between groups

Blood samples from all female pigs were collected along the study period for detection of PPV1 antibodies and PPV1 DNA by blocking ELISA (bELISA) and conventional agarose gel-based PCR, respectively. All animals from the strict control groups 3-SC and 6-SC remained seronegative to PPV1 as well as PCR negative until the study was completed (data not shown). Serology and viremia results in female pigs from Vac and NC groups in both phase I and II are depicted in Fig. 1a and b, respectively. In terms of serologic results, all animals from the groups 1-NC and 4-NC remained seronegative until challenge in each phase. In both phases, animals of vaccinated groups showed clear seroconversion 1 week after the boost immunization (SD 28). Thus, at each respective challenge day, 100% of the vaccinated animals (groups 2-Vac and 5-Vac) were seropositive for PPV1. By 2 weeks after challenge (SD 216 in phase I and SD 399 in phase II), 100% of the animals in groups 1-NC and 4-NC had seroconverted and all the animals remained seropositive until the end of the trial. Regarding PPV1 detection by PCR, no viremia was detected in any of the vaccinated animals in any phase throughout the study, before and after challenge. Oppositely, all the animals from groups 1-NC and 4-NC became PPV1 positive within 1 week after challenge.

### Fetuses evaluation

**Fig. 2:**
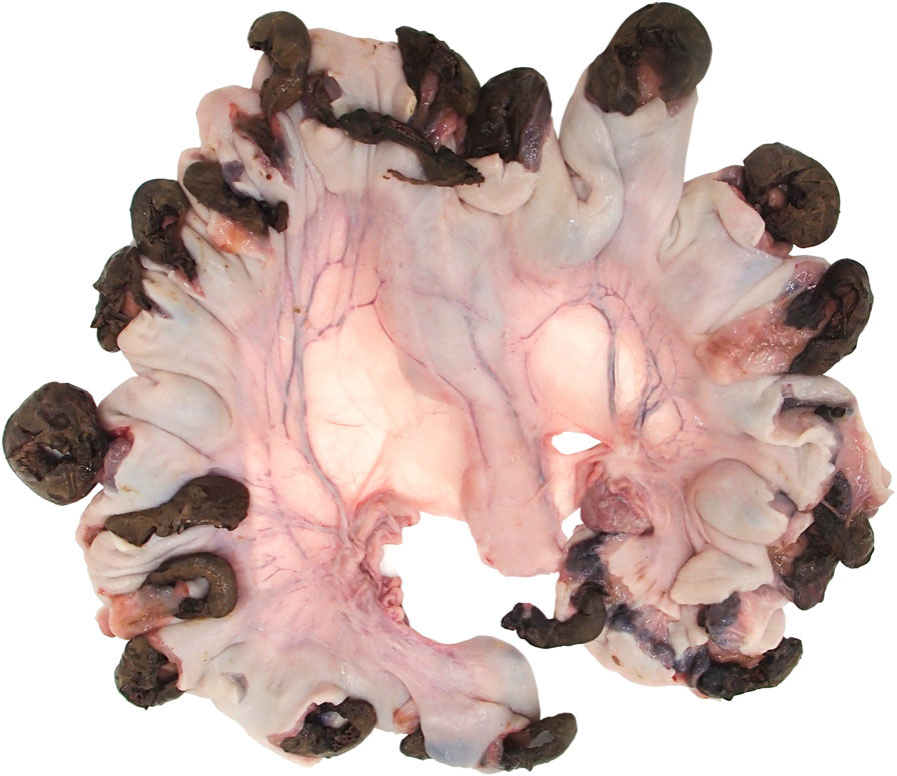
Fetuses in advanced stage of dehydration (mummification) harvested approximately at day 90 of gestation. This litter belonged to a non-vaccinated gilt that was challenged with PPV1 at approximately day 40 of gestation

Evaluation of the reproductive tract of the study animals revealed pregnancy with a variable number and condition of fetuses in most sows. Fetuses were delivered aseptically via caesarean and classified as normal, autolyzed, or mummified. Frequency of fetuses in each fetal condition category per treatment group and phase is described in Table 3. The number of fetuses per gilt was numerically higher in the vaccinated groups than in the negative control groups in both study phases. In sows from the negative control groups 1-NC and 4-NC, most litters displayed high percentages of dead (basically mummified and few autolytic) fetuses, while litters from the vaccinated groups 2-Vac and 5-Vac were predominantly normal, thus, the average fetal mortality rate was much higher in the non-vaccinated groups. Fetuses in advanced stage of dehydration (mummification) from a litter of a non-vaccinated experimentally infected gilt are shown in Fig. 2.

**Table 3:** Evaluation of fetuses at necropsy. All fetuses were analyzed for their condition and allocated to three categories: normal, autolyzed and mummified

	Group	No. of gilts	No. of fetuses	Litter size (mean)	Normal (%)	Autolyzed (%)	Mummified (%)
Phase I	1-NC	8	110	13.8	20.0	4.5	75.5
	2-Vac	10	178	17.8	97.2	0.0	2.8
Phase II	4-NC	8	86	10.8	7.0	8.1	84.9
	5-Vac	6	72	12.0	97.2	1.4	1.4

*NC* negative control; *Vac* vaccinated animals with the PPV1 subunit vaccine

### Porcine parvovirus 1 infection in fetuses

**Fig. 3:**
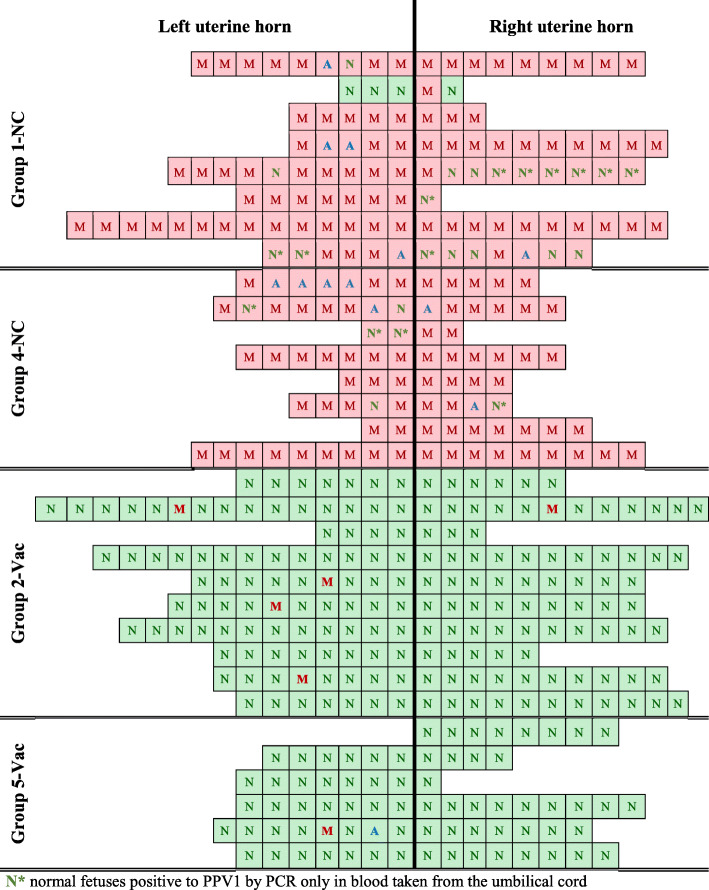
Number of fetuses per gilt and their location in the uterus together with their macroscopic condition and infectious status to PPV1. Each line represents a litter and each square a fetus. Position of the square represents the position of the fetus in the respective uterine horn. The macroscopic condition of the fetus is represented by letter N (normal), A (autolytic) or M (mummified). The squares filled in green are negative to PPV1 by PCR whereas those filled in red a PPV1 positive

To assess the PPV1 infectious status of the fetuses, umbilical cord blood, thoracic wash, and tissue (i.e. lung and kidney) samples were collected and tested by conventional agarose gel-based PCR. Porcine parvovirus 1 infection of fetuses was the primary outcome parameter for efficacy; a fetus was considered positive for PPV1 infection if it was positive by PCR in at least one of the investigated samples. The location of the fetuses in the uterus, their external condition as well as their corresponding infectious status to PPV1 is represented in Fig. 3. The percentage of PPV1 PCR-positive fetuses in phase I was 96.4% in the non-vaccinated and challenged control group 1-NC whereas 0.0% of PPV1 positive fetuses were detectable in the vaccinated group 2-Vac (*p* < 0.0001). Similarly, in the re-vaccination phase II, the percentage of positive fetuses to PPV1 by PCR was 100.0% in the non-vaccinated and challenged control group 4-NC and 0.0% in the vaccinated group 5-Vac (*p* = 0.0003).

## Discussion

Studies in the last fifteen years focused on the genetic diversity in VP1 and VP2 from field PPV1 isolates have revealed at least seven phylogenic clusters, from A to G [4]. Only a few PPV1 isolates belonging to clusters C and D (including strain 27a of which the VP2 antigen is included in ReproCyc® ParvoFLEX) have been predominant in Europe in recent years [12, 13]. The NADL2 non-virulent strain (cluster A) is currently used in several commercial inactivated vaccines and its relatively weak sequence similarity with recent field isolates from Germany has raised the hypothesis that new PPV1 strains may interfere with the efficacy of the currently used vaccines [1, 12]. For the time being, good protection after a PPV1 experimental infection has been reported even though when the vaccine strain was not closely related to the challenge strain [14]; thus, classical PPV1 vaccines are generally considered efficacious. In any case, vaccines based of more recent circulating strains deserve to be further evaluated as alternative approaches to fight against reproductive disorders caused by PPV1. Here, we report on the outcome of a DOI trial after intramuscular (i.m.) vaccination of gilts with a novel subunit vaccine against porcine parvovirosis.

Detailed requirements (e.g. Ph. Eur. monograph 0965) must be fulfilled in the EU for the development of vaccines against PPV1. These include the assessment of the DOI, which ensures that the animal is provided with a period of protective immunity that encompasses key time windows crucial for preventing infection and, in consequence, reproductive failure. Study validity as well as vaccine efficacy were demonstrated as: (i) neither PPV1-DNA nor PPV1-antibodies could be detected by PCR or bELISA in serum samples of strict control animals and negative control animals prior to challenge, (ii) 96.4 to 100% of fetuses in the negative control groups were PPV1 positive by PCR and (iii) 100% of fetuses in the PPV1 vaccine groups were PPV1 negative by PCR. Since protection beyond 6 months post-vaccination was not assessed, re-vaccination at regular intervals every 6 month should be recommended when using the present PPV1 subunit vaccine. Regarding the design of the study, a non-vaccinated non-challenged group was not included, as safety evaluation of repeated doses of the present PPV1 subunit vaccine in bred pigs and in offspring has been already assessed under experimental settings [15].

Porcine parvovirus 1 viremia after challenge was detectable only in non-vaccinated gilts belonging to the negative control groups, demonstrating that vaccination can prevent viremia, which is consequently related to prevention of the PPV1 to cross the placenta and infect the developing fetuses. In former experiments, low antibody titers to PPV1 were detected in umbilical blood of fetuses from sows vaccinated with inactivated PPV1 vaccines [7, 16]. It was discussed whether this observation should be interpreted as virus replication in immune-competent fetuses or whether it represents an euthanization artefact, resulting from contamination of fetal blood with maternal blood. In the present work, however, PPV1 presence in fetuses from vaccinated sows was not observed as neither umbilical blood nor thoracic wash samples were positive to PPV1 DNA evaluated by PCR. The latter was also confirmed clinically, as fetal deaths caused by PPV1 infection were prevented in all vaccinated groups at day 90 of gestation.

Beside the mummified fetuses of the control groups, gilts did not show clinical signs attributable to infection or vaccination. On average, the litter size was numerically larger in gilts from the vaccinated groups than from the negative control groups in both study phases. PPV1 infection early in gestation (until approximately day 35) results in embryonic death and maternal resorption of fetal tissues, which is clinically manifested as a reduction in the litter size [1]. In this study, animals were challenged at day 40 of gestation, thus, the smaller litter size in the non-vaccinated gilts is difficult to be merely explained by the infection with PPV1, especially when many factors are known to influence this parameter [17]. More salient is the fact that the mortality rate among the fetuses of the vaccinated and non-vaccinated sows differed significantly: whilst around 80% of the fetuses from the negative control groups were dead and showed various degrees of fetal mummification, such pathological conditions were observed in less than 3% of the fetuses in both vaccine groups. In addition, no PPV1- DNA could be detected in the fetuses of both vaccine groups. Fetal mummification has been linked to parity, litter size, uterine capacity, temperature of the environment, presence of mycotoxins, and infectious diseases [18]. Since PPV1 infection was discarded in the abnormal fetuses from vaccinated sows, another etiology was likely involved. Stillborn piglets are an important clinical feature associated with PPV1 infection. Some stillbirth originates before the onset of parturition, including mummified fetuses and autolytic stillborns. Nonetheless, the majority of stillborns die intrapartum [19]. Whether stillbirths may have varied between vaccinated and non-vaccinated female pigs could not be properly addressed in this study, as fetuses were delivered via caesarean at day 90 of gestation.

Many immunological studies have proved that the presence of neutralizing serum antibodies in the dam is a decisive factor in the outcome of the PPV1 infection; they prevent fetal death by avoiding the virus to cross the placenta barrier [7–9]. In fact, genetically modified Lactobacillus and Pseudorabies virus expressing the VP2 protein of PPV1 were shown to induce neutralizing antibodies against PPV1 [10, 11], but in vivo challenge experiments for testing vaccine efficacy were missing so far. Herein, the presence of PPV1-specific neutralizing antibodies was not assessed, which would have been useful to confirm that the detected antibodies by bELISA were neutralizing antibodies. However, it is worth mentioning that the proportion of seropositive gilts to PPV1 increased steeply after the second dose vaccination in phase I and after re-vaccination in phase II. Outstandingly, the proportion of seroconverted gilts increased reaching 100% by 6 month after the first and second booster dose in phase I and II, respectively. The later ensured that the totality of animals in the vaccinated groups tested seropositive at challenge. These, indeed, remained seropositive until euthanized at day 90 of the gestation period.

The DOI of a vaccine against PPV1 is specifically linked to the protection of fetuses during the gestational period when following the recommended vaccination schedule. An ideal vaccine would achieve a long DOI to reduce the risk of infection as well as re-infection with PPV1 during an animal’s lifetime. Simultaneously, it must effectively reduce viremia and clinical signs. In this study, compliance of the innovative PPV1 subunit vaccine with the immunogenicity requirements of the Ph. Eur. was demonstrated. Considering the absence of PPV1 in fetuses, the level of protection in all vaccinated groups was 100% (both after basic vaccination and re-vaccination). In summary, the PPV1 subunit vaccine can help limit the acute economic losses caused by PPV1 infection and, at the same time, its effectiveness against heterologous PPV1 strains supports the fight against different PPV1 field isolates. Notwithstanding, extrapolation of results obtained under controlled conditions to the field should be done very carefully, as vaccine outcomes might be affected by several factors masked at experimental level (e.g. infection pressure on the farm, co-infection with other swine pathogens, management practices and housing conditions). In addition, it is important to keep in mind that the challenge with PPV1 was performed at day 40 of pregnancy, hence, whether the outcome of this study would have been different upon challenge earlier or later on during gestation cannot be discarded.

## Conclusion

It is concluded that vaccination with the novel PPV1 subunit vaccine, which is based on the VP2, prevents PPV1 viremia and protects fetuses against heterologous PPV1 challenge with a DOI of at least 6 months after vaccination. Furthermore, a DOI of 6 months and the prevention of viremia was also confirmed after a re-vaccination with a single dose, 6 months after the initial dosing scheme. This vaccine is appropriate for protection against disease in a heterologous challenge scenario and its potential use in mass vaccination protocols advocate ReproCyc® ParvoFLEX as a real and competitive alternative to the classical PPV1 inactivated vaccines.

## Methods

### Animals

Forty-six mixed breed nulliparous gilts of approximately 5 months of age were obtained commercially from a nucleus herd (German Federal Hybrid Breeding Program [BHZP GmbH]) with a high health status including vaccination against porcine circovirus type 2 (PCV2). Most importantly, animals were not vaccinated against and tested free (ELISA and PCR) for porcine reproductive and respiratory syndrome virus (PRRSV) and PPV1 prior to the start of the study. The study was conducted in the animal facility of Boehringer Ingelheim Veterinary Research Center (BIVRC) GmbH & Co. KG (Hannover, Germany). Animals were transported to the center 8 days prior to the first vaccination (SD 0). All study animals were housed in facilities appropriate for their age and kept under controlled conditions according to BIVRC standards. Water and feed were available ad libitum in appropriate quality and were managed in accordance with the pig’s requirements at its age. During housing, animals were monitored daily for health status. All procedures were carried out under approval of the Ethical Committee of the LAVES organization (Lower Saxony State Office for Consumer Protection and Food Safety) and in accordance with GCP, VICH Guideline GL9, CVMP/VICH/595/98-Final; Directive 2001/82/EC (as amended in 2009), EMA/CVMP/IWP/594618/2010 and Ph. Eur. Monograph 0965. All sections of this report adhere to the ARRIVE Guidelines for reporting animal research [20]. A completed ARRIVE guidelines checklist is included in Checklist S1.

### Study design

The study had a blinded, randomized, negative-controlled design with six treatment groups (Table 1). It was conducted in two phases: the first phase evaluated the DOI of the basic vaccination scheme (6 months after a two-dose regimen) and the second phase assessed the DOI of the re-vaccination (6 months after a single boost immunization). All animals were housed together in group-housing systems until challenge. At challenge, animals from phase I were separated from the ones belonging to the re-vaccination phase II. Following guidance of Ph. Eur. Monograph 0965, a minimum of 7 animals in the vaccinated and 5 animals in the control groups were enrolled into the study. Hence, a total of 46 gilts were randomly assigned to one of the treatment groups by drawing lots. The negative control groups received placebo, i.e. sodium chloride (NaCl), twice in a 3-week interval (SD 0 and 21) in each respective phase (group 1-NC in phase I and group 4-NC in phase II). At the same time points, groups 2-Vac (phase I) and 5-Vac (phase II) received the PPV1 subunit vaccine whereas the strict control groups (3-SC and 6-SC in phase I and II, respectively) received no treatment. In phase I, all gilts were estrus synchronized and artificially inseminated by 4 months from the last day of treatment (SD 21). Animals from groups 1-NC and 2-Vac were challenged at day 40 (± 1) of gestation. Necropsy was performed at day 90 (± 1) of gestation thereby collecting the fetuses for evaluation and sampling. To evaluate the DOI of the re-vaccination scheme (phase II), female pigs in group 5-Vac were re-vaccinated 6 months after the basic vaccination (SD 202) with a single dose of the PPV1 subunit vaccine while those belonging to the group 4-NC received placebo (NaCl solution). Females were estrus synchronized and artificially inseminated about 4 months from re-vaccination and they were challenged at day 40 (± 1) of gestation while their fetuses were collected at day 90 (± 1) of gestation. For blinding purposes, the groups were named using capital letters and the respective investigators were not informed about the group treatments behind the blinding code. In addition, the person administering the vaccine and placebo was not the same person responsible for the clinical observation and sampling of the animals. The pathologist as well as all technical staff involved in the necropsies were also blinded for allocation of the animals to the treatment groups. The blinding code was revealed ad the end of the study after data base hard lock.

### Vaccine and control product administration

The vaccine was prepared at Boehringer Ingelheim Vetmedica Inc. (BIVI; St. Joseph, MO, USA) for experimental use only; all batches were manufactured using the highest seed passage level for production of the vaccine and this was formulated to contain the minimum antigen content of 1 μg of VP2-protein of the PPV1-27a isolate [12] per 2 ml dose. ReproCyc® ParvoFLEX also contains carbomer as adjuvant and NaCl solution for injections as excipient. Therefore, 0.9% NaCl solution for injection was used as a control product (placebo). Both the vaccine and placebo were injected i.m. twice (2 ml dose) 3 weeks apart. On SD 0, treatments were administered in the right side of the neck and on SD 21 in the left side of the neck for groups 1-NC, 2-Vac, 4-NC, and 5-Vac. At re-vaccination (phase II), groups 4-NC and 5-Vac were injected once i.m. with 2 ml in the right side of the neck.

### Animal estrus synchronization and insemination

A formerly described protocol for fixed-time ovulation and insemination was followed after approximately 4 months from the last day of treatment in both phase I and II [21]. Hormone injections were performed i.m. in a different location than the vaccine and all drugs were administered according to manufacturers’ recommendations. Artificial insemination was performed at least twice if possible after the injection of the last hormonal treatment (Ovogest®, MSD Animal Health, Luzern, Germany). Semen was sourced from an approved boar stud (GFS Top Genetik, Rohrsen, Germany) and it was tested free from PPV1, PCV2 and PRRSV. Pregnancy checks were performed by ultrasound investigation prior to challenge, so as gilts that were not pregnant were removed from the study. These gilts were euthanized by exsanguination after electrical stunning and submitted for necropsy evaluation when possible; a macroscopic inspection of the carcass was performed.

### Challenge material characteristics and administration

The challenge material was prepared prior to the challenge event by the BIVRC laboratories. The challenge heterologous PPV1 strain, PPV1 EU strain 401/09 (198669), was obtained from the *Universität Leipzig* (Leipzig, Germany) and was stored frozen at ≤ − 70 °C until used. Two different challenge batches were handled during the study: batch H96–064 was used in phase I and batch H183–064 in phase II. Prior to challenge, the stored material was thawed and diluted in Dulbecco’s Modified Eagle’s Medium. The titer administered was of 6.0 log_10_ tissue culture infective dose 50 (TCID_50_) per dose for batch H96–064 and 7.3 log_10_ TCID_50_ per dose for batch H183–064. The titer adjusted challenge material was held refrigerated until its administration to the animals. All animals challenged were inoculated with 2 ml of challenge material per each nostril and an additional 2 ml i.m. in the right side of the neck, resulting in a total of 6 ml of challenge material administered.

### Variables for efficacy assessment

The evaluation of the study was performed according to Ph. Eur. monograph 04/2013:0965, which states that the test is valid if: not fewer than 90% of piglets from control gilts (groups 1-NC and 4-NC) are infected with PPV1 and the average number of piglets per litter from vaccinated gilts (groups 2-Vac and 5-Vac) is not fewer than 6. Consequently, the primary efficacy outcome variable for the study was PPV1 infection of fetuses as determined by PCR; the vaccine was considered efficacious (i.e. the DOI at 6-month post-vaccination or re-vaccination was achieved) if ≥80% of fetuses in each of the vaccinated groups (2-Vac and 5-Vac) were negative for PPV1 by PCR in all the investigated tissue samples. Secondary efficacy outcomes included qualitative post-challenge viremia, serological status in gilts and general condition of fetuses. The validity of each of the two challenges (phase I and II) was assessed independently from each other. Thus, the test was valid as all above mentioned requirements were satisfactory fulfilled in both challenge periods.

### Gilt blood sampling

Blood samples from all gilts were collected for detection of PPV1 antibodies and PPV1 DNA. From the start of the study (SD 0), blood samples were collected weekly (SD 7, 14, 21, 28, 35) and then monthly (SD 65, 97 and 125) until phase I challenge (SD 202). After the first challenge, a similar weekly sampling routine was followed (SD 209, 216, 223) until necropsies of phase I. In phase II, the animals continued to be sampled monthly (SD 252, 283, 314, 339) until challenge (SD 385), from where they were sampled weekly (SD 392, 399, 406) until necropsies. Five ml of blood was collected per pig and sampling time point. Blood was processed for serum and aliquoted into appropriate tubes and held at − 20 °C (+/− 5 °C) before testing.

### Necropsy and fetal evaluation

All gilts were euthanized by exsanguination after electrical stunning and submitted for necropsy approximately 50 days after challenge (day 90 of gestation), ensuring BIVRC standards. Gilts were left to exsanguinate enough time to ensure death of those fetuses that could be alive. During necropsy, the reproductive tract of the gilts was removed, and the following data was recorded: number of fetuses per gilt, position of each fetus in the uterus and fetal condition. Fetuses were delivered aseptically via caesarean and numbered according to their position in the uterus starting at the left ovary through the left uterine horn, uterine body, and right uterine horn. Afterwards, the fetuses were classified as normal, autolyzed, or mummified.

### Samples to assess the infectious status of the fetuses

#### Umbilical cord blood

If possible, blood from the umbilical cord was sampled from normal fetuses. For this purpose, the umbilical cord was clamped off at the uterus and cut so that the blood could be collected from the fetal side of the cord. Umbilical cord blood samples were processed for serum and stored at − 70 °C (+/− 5 °C) before performing PPV1 DNA detection test.

#### Thoracic wash

If possible, a thoracic wash sample was collected from normal fetuses using separate equipment for each individual animal. Briefly, 3 ml of sterile phosphate-buffered saline was injected into the unopened thoracic cavity with a sterile needle and syringe, and as much fluid as possible was aspirated back into the syringe and injected into a collecting tube. Thoracic washes were stored at − 70 °C (+/− 5 °C) before testing for detection of PPV1 DNA.

#### Tissue samples

If possible, tissue samples of lung and kidney were collected separately from each mummified or autolyzed fetuses, where collection of thoracic wash and umbilical blood was not possible, using disinfected equipment for each individual animal. If any of the normal fetuses was too small at necropsy for obtaining umbilical cord blood or thoracic wash, tissue samples of lung and kidney were also collected. New sterile instruments were used for each litter/uterus. If samples could not be collected due to the desiccation of the fetus, a variety of soft tissues were collected (preferably lung and/or kidney as far as they were identifiable). Tissues were stored at − 70 °C (+/− 5 °C) before testing for detection of PPV1 DNA.

### Laboratory methods

Sera from blood samples of the gilts were investigated by bELISA for the presence of PPV1 antibodies (INgezim® PPV Compac, INGENASA, Spain). This commercially available test was used and interpreted according to the manufacturer’s recommendations. Furthermore, sera from gilts, fetal thoracic washes, umbilical cord blood samples, and fetal tissue samples were analyzed qualitatively by modifying an earlier described conventional PCR for the presence of PPV1 genetic material [22]. Briefly, 8 μL of the DNA preparation was used as PCR template and amplification was performed in a final volume of 50 μL. The reaction mixture consisted of 0.2 μM of each primer, 0.2 mM of each nucleotide, 1 × PCR buffer (QIAGEN, Hilden, Germany) and 2.5 U of *Taq* DNA polymerase (QIAGEN). RNase-free water (34.5 μL; QIAGEN) was added to prevent evaporation of the reaction mixture. The reaction was performed in a thermocycler under the following conditions: initial heating at 94 °C for 5 min and 38 cycles, denaturation at 94 °C for 30 s, annealing at 55 °C for 30 s, and extension at 72 °C for 45 s. Ten μL of the amplified product (158 base pairs) were collected and directly analyzed on an agarose gel by electrophoresis. The sensitivity and specificity of this technique was previously assessed by means of testing different samples (e.g. serum, thoracic washes) containing defined amounts of PPV1; the limit of detection obtained was at least 2.09 log_10_/ml of PPV1.

### Statistical analysis

The statistical analyses and data summaries were done using SAS software version 9.2 or a higher version (SAS Institute Inc., Cary, NC, USA). All data were summarized descriptively (sample size “n”, minimum, maximum, median, mean, confidence interval, standard deviation and/or frequencies) based on the type of variable and analyzed assuming a completely random design structure. The strict control groups 3-SC and 6-SC were not evaluated statistically. All tests on differences between vaccinated (2-Vac and 5-Vac) and negative control (1-NC and 4-NC) groups were designed as two-tailed tests. For all tests, differences were considered to be statistically significant only if *p* ≤ 0.05. Briefly, Fisher’s exact tests on differences between vaccinated groups and the respective control groups in each phase were used to evaluate proportion of gilts with positive PPV1 PCR and bELISA results in blood samples. Also, an analysis of variance using the Tukey–Kramer test was used for mean comparison of blocking percentages between groups. Lastly, Wilcoxon Mann-Whitney test was used to assess proportion and number of fetuses per gilt and treatment group with positive PPV1 PCR samples.

## Data Availability

The data that support the findings of this study are available from Boehringer Ingelheim Animal Health, Inc. but restrictions apply to the availability of these data, which were used under license for the current study, and so are not publicly available. Data are however available from the authors upon reasonable request and with permission of Boehringer Ingelheim Animal Health, Inc.

## Abbreviations

DOI: Duration of immunity
ELISA: Enzyme-linked immunosorbent assay
GCP: Good Clinical Practice
i.m.: Intramuscular
NC: Negative control
PCR: Polymerase chain reaction
Ph. Eur.: *European Pharmacopoeia*
PPV1: Porcine parvovirus 1
PRRSV: Porcine reproductive and respiratory syndrome virus
SC: Strict control
SD: Study day
TCID_50_: Tissue culture infective dose 50
Vac: Vaccinated
VP: Viral protein
